# *Euterpe oleracea* fruit (Açai)-enriched diet suppresses the development of experimental cerebral malaria induced by *Plasmodium berghei* (ANKA) infection

**DOI:** 10.1186/s12906-021-03495-9

**Published:** 2022-01-11

**Authors:** Karen Renata Herculano Matos Oliveira, Marjorie Lujan Marques Torres, Nayara Kauffmann, Brenda Jaqueline de Azevedo Ataíde, Nívia de Souza Franco Mendes, Larissa Medeiros dos Anjos, Rosivaldo dos Santos Borges, Carlomagno Pacheco Bahia, Luana Ketlen Reis Leão, Adelaide da Conceição Fonseca Passos, Anderson Manoel Herculano, Evander de Jesus Oliveira Batista

**Affiliations:** 1 Laboratory of Experimental Neuropharmacology, Biological Science Institute, UFPa, Belém, PA Brazil; 2 Laboratory of Protozoology, Topical Medicine Nucleus, UFPa, Belém, PA CEP: 66055-240 Brazil; 3 Laboratory of Pharmaceutical Chemistry, Health Science Institute, UFPa, Belém, PA Brazil; 4 Laboratory of Neuroplasticity, Health Science Institute, UFPa, Belém, PA Brazil

**Keywords:** *Euterpe oleracea*, Açai, natural product, Cerebral malaria, *Plasmodium*, Neurobehavioral impairment, Blood-brain barrier

## Abstract

**Background:**

Cerebral malaria is one of the most severe complications attributed to protozoal infection by *Plasmodium falciparum*, gaining prominence in children mortality rates in endemic areas. This condition has a complex pathogenesis associated with behavioral, cognitive and motor sequels in humans and current antimalarial therapies have shown little effect in those aspects. Natural products with antioxidant and anti-inflammatory properties have become a valuable alternative therapeutic option in the treatment of distinct conditions. In this context, this study investigated the neuroprotective effect of *Euterpe oleracea* (açai) enriched diet during the development of experimental cerebral malaria induced by the inoculation of Swiss albino mice with *Plasmodium berghei* ANKA strain.

**Methods:**

After *Plasmodium* infection, animals were maintained on a feeding with *Euterpe oleracea* enriched ration and parameters such as survival curve, parasitemia and body weight were routinely monitored. The present study has also evaluated the effect of açai-enriched diet on the blood-brain barrier leakage, histological alterations and neurocognitive impairments in mice developing cerebral malaria.

**Results:**

Our results demonstrate that between 7th–19th day post infection the survival rate of the group treated with açai enriched ration was higher when compared with *Plasmodium-*infected mice in which 100% of mice died until the 11th days post-infection, demonstrating that açai diet has a protective effect on the survival of infected treated animals. The same was observed in the brain vascular extravasation, where Evans blue dye assays showed significantly less dye extravasation in the brains of *Plasmodium-*infected mice treated with açai enriched ration, demonstrating more preserved blood-brain barrier integrity. Açai-enriched diet also attenuate the histopathological alterations elicited by *Plasmodium berghei* infection. We also showed a decrease of the neurological impairments arising from the exposure of cerebral parenchyma in the group treated with açai diet, ameliorating motor and neuropsychiatric changes, analyzed through the SHIRPA protocol.

**Conclusion:**

With these results, we conclude that the treatment with açai enriched ration decreased the mortality of infected animals, as well as protected the blood-brain barrier and the neurocognitive deficits in *Plasmodium*-infected animals.

## Background

Central nervous system (CNS) is often a common target to a number of tropical infectious diseases, including malaria, which is caused by infection with *Plasmodium* parasite and represents one of the world-leading causes of disease-related mortality in tropical regions [1, 2]. Clinical signs can vary from asymptomatic parasitaemia to a severe and fatal condition [3, 4]. Cerebral malaria (CM) is the most severe complication resulting from *Plasmodium falciparum* infection with a mortality rate of more than 25% of the cases [5, 6]. When untreated, patients present long-term neurological deficits which include cognitive and motor dysfunctions.

CM surviving children exhibit extensive neurocognitive impairments after recovering from the acute phase of the disease. Tasks such as attention, working memory and learning are clearly compromised in children [7, 8]. Movement disorders such as ataxia, tremors and dystonia were also detected [9, 10]. The mechanisms involved in these impairments are still unknown, although pathological events associated to CM strongly suggest the involvement of intense cerebral hemorrhaging and blood-brain barrier (BBB) disruption in those events [11, 12].

Murine model of CM with *Plasmodium berghei* ANKA infection displays several neurological features of human CM (HMC) and therefore may be a valuable tool to identify potential new candidates for adjunctive therapies in the human disease. Experimental cerebral malaria (ECM) can be assessed by using rodent models with susceptible mouse strains as C57BL/6 and Swiss albino mice infected with *Plasmodium berghei* ANKA parasites [13, 14]. Intense pro-inflammatory response, vascular obstruction and the breakdown of blood-brain barrier are the main and key pathological features observed both in HCM and ECM.

The search for new therapeutic approaches represents a new challenge since the increased number of patients resistant to now available drugs. In this context, the study and use of natural products that can attenuate or suppress neurological deficits associated with the disease has been growing in this scenario.

*Euterpe oleracea* (EO) is a large palm tree of the Arecaceae family found in native forests of Brazilian Amazon. Fruit pulp, known popularly as açai or juçara, is widely consumed by the Amazonian population and is also used to prepare energy drinks and dietary supplements with increasing nutraceutical potential [15, 16]. Bromatological analyses have shown that açai present high nutritional values with elevated concentrations of antioxidants such as tocopherol and polyphenolic compounds as anthocyanins and flavonoids [17, 18]. Medicinal and pharmacological properties of the fruit have already been reported in several experimental studies showing remarkable antioxidant, anti-inflammatory and antimicrobial effects [16, 19–21].

Recently, açai methanolic extract demonstrated an antimicrobial effect against clinical strain of *Staphylococcus aureus*, showing synergistic effect with classical antimicrobial drugs [22]. Also, a study demonstrated the anti-leishmanial activity of EO treatment without any cytotoxic effect in the murine host cell [23]. In CNS, the treatment with açai evokes a remarkable anticonvulsivant effect, protecting against seizures and electrocortical alterations [20].

Although a number of studies described the protective effect of açai, no systematic research points out the potential protective effects of the fruit in neurological disorders induced by infectious diseases. In this context, the aim of the present study was to investigate whether an açai-enriched diet is able to prevent the outcome of CM and the neurobehavioral alterations induced by the disease.

## Methods

### Animals

Healthy Swiss albino mice aged 5–6 weeks and weighing 25-30 g were obtained from local Institutional (Federal University of Para) Animal Care Facilities. Male and female mice were used in the study once both develop a similar neurological response pattern to malaria infection. Animals were housed in groups of three to five per cage in pathogen-free conditions and controlled room temperature (23 ± 3 °C). Filtered water and standard food was offered ad libitum until the beginning with *Euterpe oleracea* diet. The study was approved by the Animal Ethics Committee_Federal University of Para under the protocol number 6211241117 and was carried out in accordance with the guidelines for the ethical use of laboratory animals (Animal Ethics Committee_Federal University of Para). The work has also been reported in accordance with the ARRIVE guidelines (Animals in Research: Reporting In Vivo Experiments). All the plant collections were conducted in accordance to standard protocols and were properly authorized. All efforts were made to minimize potential suffering and to reduce the number of animals used in all experiments.

### Preparation of *Euterpe oleracea*-enriched ration

Fruits from *Euterpe oleracea* were obtained from a local farm (Santa Fé) at Santa Izabel County (Para state, Brazil) and plant species were properly identified. Fresh açai pulp was processed from the raw fruits present in the clusters of palm trees. The protocol used to prepare açai pellets was adapted from Brasil et al., [15]. Briefly, açai-enriched ration (AER) was produced by mixing standard commercial feed (Nuvital) with the açai pulp in the proportion of 10:1 (g/g) to produce the pellets. These pellets were slightly heated to remove humidity, weighed and stored in hermetically sealed plastic bags and kept under refrigeration for periods not exceeding 3–5 days.

### Infection with *plasmodium berghei* ANKA strain and experimental groups

Mice were infected with *Plasmodium berghei* ANKA (PbA) strain using the protocol previously described by Oliveira et al. [24] Briefly, the animals received an intraperitoneal (i.p) injection of 10^6^ parasitized erythrocytes, suspended in 0.1 mL of phosphate-buffered saline (PBS). Control animals received the same volume of PBS. Body weight variation and survival rates curve were evaluated throughout the experimental period. The evolution of the infection was also monitored daily by determining the parasitemia by Giemsa-stained slides obtained from mice tail blood as described by Ataide et al. [25]. Animals positive to CM show symptoms as poor reflexes, roll-over, ataxia, limb paralysis, seizures and coma between 6- and 10-days post infection. All observations were conducted using a double-blind assessment.

**Fig. 1:**
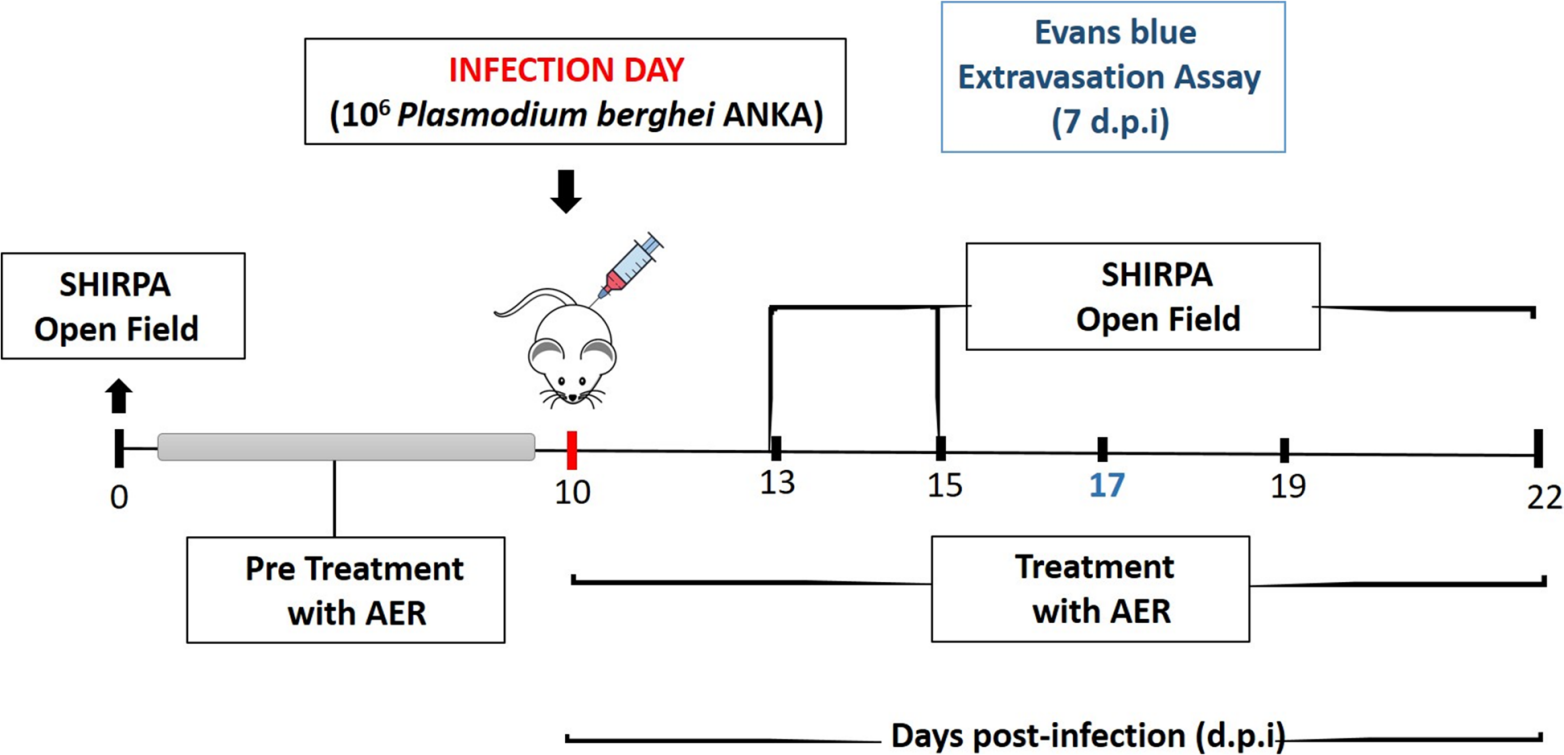
A schematic representation of the experimental timeline. Swiss albino mice were fed with açai enriched ration for 11 days before the inoculation with *Plasmodium berghei* ANKA strain (PbA) and continued with the diet until the last day of experiment. After the inoculation with 10^6^ parasitized erythrocytes, the animals from distinct groups were submitted to the biochemical and neurobehavioral analysis

To evaluate the effect of açai-enriched diet in the clinical progression of CM, animals were first fed with either standard commercial ration (*n* = 17) or AER (*n* = 17) 10 days prior to PbA infection until the last day of the experiment. At day 11, mice from both control (standard ration) and AER group were reassigned to either non-infected (control) or PbA-infected groups. All the experiments were repeated at least three times (Fig. 1).

### Brain vascular permeability assay

To evaluate brain vascular leakage Evans blue dye extravasation was measured as previously described by Kim et al. [26]. On 7th day post-infection, mice from all experimental groups were anesthetized with ketamine/xylazine solution (1:1 mg/kg; i.p) and transcardially injected with 2% Evans Blue solution (EB) for approximately 5 min. Animals were then perfused with saline solution for 30 min through the left ventricle and the whole brains removed. Dye was extracted from the brain with dimethyl formamide for 48 h at 37 °C (in the dark). Formamide concentration was quantified spectrophotometrically at 620 nm, and results normalized to the brain dry weight.

### Brain histological analysis

For histological evaluation, mice brains of each experimental group were carefully dissected, fixed in 10% formalin and embedded in paraffin wax. Serial coronal sections into 5 μm were performed using microtome and stained with hematoxylin-eosin (HE) according to standard procedures. The brain cortex was examined in the cortical-medullary area under a light microscope and photographed at 10x and 40x objective lens.

### Open field test

The open field test is often used to assess the locomotor ability in rodents. The test was applied at the 3rd, 5th, 7th, 9th, and 12th day post-infection and consists of placing the animal at the center of a circular box (Ø = 82 cm x h = 52 cm) with a square grid marked on its floor. The animal movement among the grid squares was recorded for 5 min and data processed and analyzed using the Debut Video Capture software, version 1.49, and the X-PloRat software. The values were expressed as the number of squares crossed per minute.

### Neurobehavioral assessment

SHIRPA (SmithKline Becham Pharmaceuticals; Harvwell, MRC Mouse Genome Centre and Mammalian Genetics Unit; Imperial College School of Medicine at St. Mary’s; Royal London Hospital, St. Bartholomew’s and the Royal London School of Medicine; Phenotype Assessment) behavioral protocol was used to evaluate neurological alterations as described by Martins et al. [27] SHIRPA protocol consists of behavioral and functional parameters used to determine neurological impairment in murine rodents. Parameters were grouped into four main categories: neuropsychiatric aspects (transfer arousal, touch escape, positional passivity, provoked biting, aggression, vocalization, fear, irritability), reflex and sensorial function (visual position, pinna reflex, corneal reflex, toe pinch, straightening reflex), muscle tone and strength (abdominal tone, limb tone, grip strength) and motor activity (locomotor activity, gait, wire handling, negative geotaxis, pelvic elevation, tail elevation, body position tremor). Each parameter was scored (the scores vary according to the evaluated parameter, being able to switch between score 0, meaning absence or inability to react; and score 6, of extreme excitability) to provide a quantitative analysis of impairments during the course of the disease. SHIRPA protocol was performed before infection with PbA to record each animal baseline and on days 3, 5, 7, 9 and 12 post-infection. All these parameters were performed using double-blind analysis.

### Statistical analyses

The data was expressed as means ± standard deviation. Survival data were compared and analysed using log-rank test of Kaplan-Meier curves. Differences between groups were evaluated using a one-way ANOVA, followed by Tukey’s post-test. These analyses were performed in GraphPad Prism Software and *p* values less than 0.05 were considered significant.

## Results

### *Euterpe oleracea*-enriched diet suppresses the development of CM and the breakdown of BBB in *P. berghei* ANKA infected mice

*To evaluate the effect of açai-enriched diet on the outcome of CM, albino* Swiss *mice were infected with PbA* strain which consists in a well-established model of CM that reproduces most features of the human *P. falciparum* infection. All CM signs were observed between day 6 to 12 post-infection. All the animals of PbA-control (standard diet) group presented signs as poor reflexes, roll-over, ataxia, limb paralysis, seizures and coma. None of these neurological signs were observed in the PbA-infected group fed with açai-enriched diet, suppressing the development of CM in mice.

**Fig. 2:**
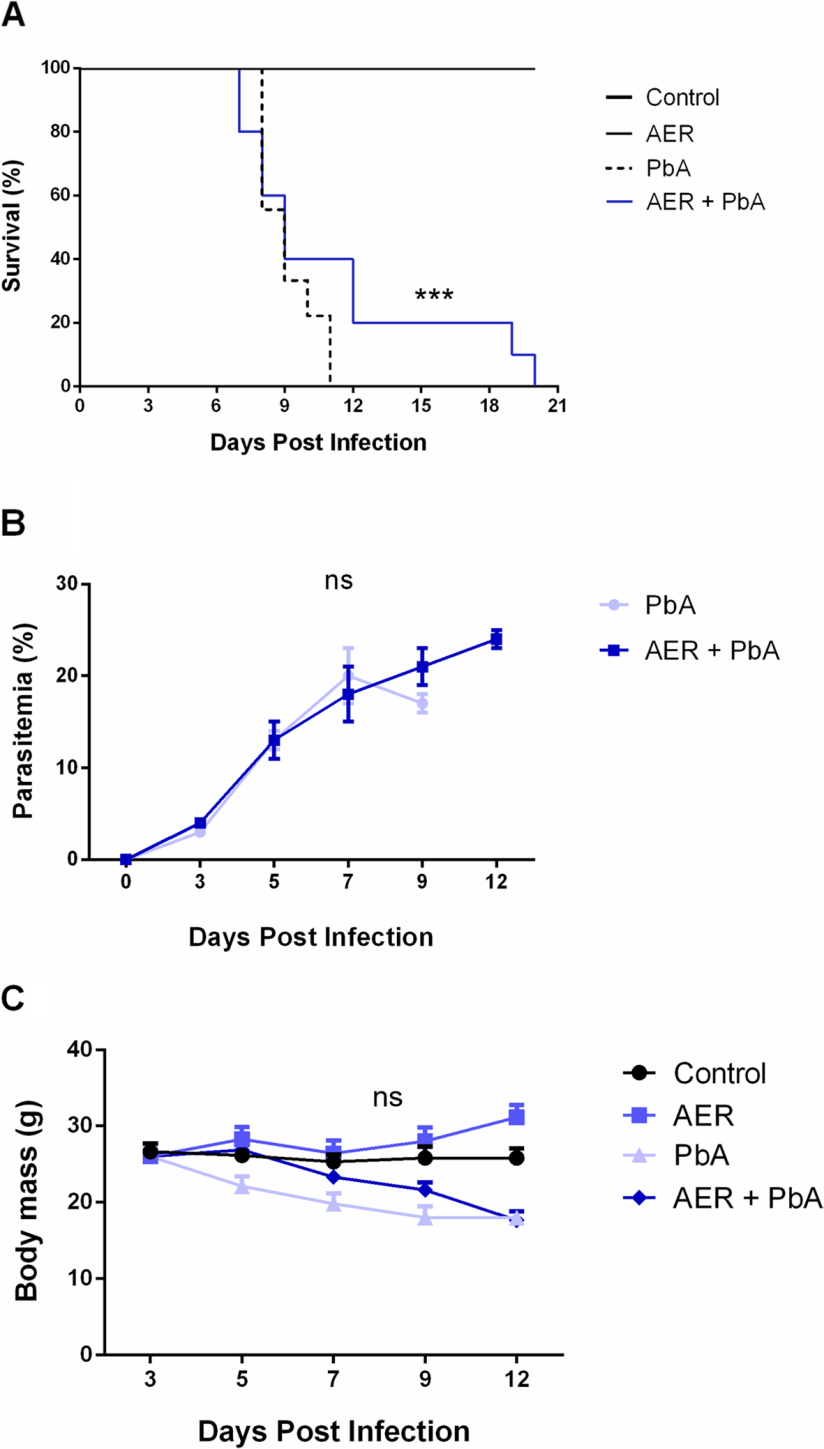
**A** Survival curve (**B**) Parasitemia and (**C**) variation in body mass of swiss albino mice infected with *Plasmodium berghei* ANKA strain (PbA) and treated with açai enriched ration (AER). Animals in the experimental groups (*n* = 17 animals/group) were divided into four groups: Control group received only an injection of phosphate-buffered saline; AER group received only the treatment with açai-enriched diet; PbA group were inoculated with 10^6^ parasitized erythrocytes and PbA + AER group were inoculated with PbA strain and received açai-enriched diet; (*n* = 17, ***p < 0,01 vs PbA)

Infection with PbA induced a fast and progressive decrease in the survival of animals fed with the standard diet (Fig. 2A). As we can observed in Fig. 2A, at 7th day post-infection (d.p.i.), the survival rate of PbA-infected mice fed with standard diet decreased 40%, increasing to 50% by the 8th d.p.i., 70% by the 9th d.p.i. and 80% by the 10th d.p.i. On the 11th day post-infection, PbA group reached 100% of mortality (Fig. 2A). This death pattern contrasted with observed in the PbA group fed with the açai-enriched diet, once at 9th d.p.i. the survival rate stabilizes at 50%. At 11th d.p.i. 40% of the PbA-infected mice fed with açai-enriched diet survived and died between the 15th d.p.i. with no neurological signs of CM (Fig. 2A).

PbA-infected mice also showed a rapid increase in parasitemia between days 3 and 5 post infection, keeping low and stable until 12 days post infection, which is characteristic of the disease progression. Data also showed that açai-enriched diet did not alter the evolution of parasitemia in animals infected with PbA (Fig. 2B). Additionally, body mass was not modified by the treatment with açai-enriched diet in none of experimental groups (Fig. 2C).

**Fig. 3:**
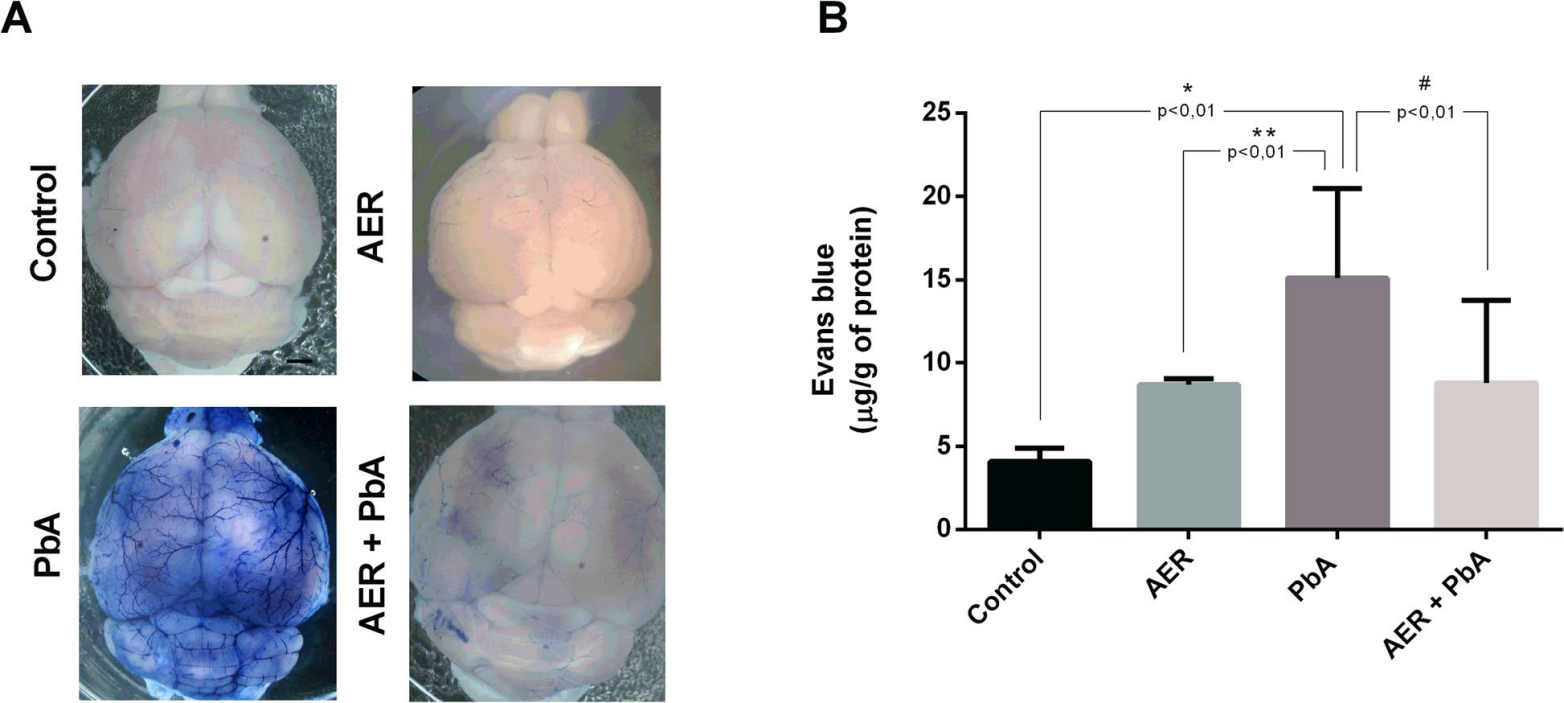
Treatment with açai enriched ration maintains blood-brain barrier integrity in ECM. **A** Qualitative analysis of the whole brain after Evans Blue dye administration in uninfected control group, AER group, PbA-infected mice and PbA + AER group; **B** Quantification of Evans blue (EB) extravasated into brain at 7 days post-infection. The experiment was repeated three times and subjected to one-way ANOVA and expressed as mean ± SD; (*n* = 17, *p < 0,01 vs control; ^#^*p* < 0,01 vs PbA)

In CM condition, the neurological signs were also accompanied by the breakdown of BBB. To assess the effect of açai-enriched diet in the integrity of BBB, Evans Blue dye extravasation was measured in the brain of mice infected with PbA. At 7 days post infection, the brain of PbA-infected animals showed remarkable extravasated vessels (26 μg/g of Evans blue) when compared to the PbA-infected mice fed with açai-enriched diet (12 μg/g of Evans blue); (Fig. 3) demonstrating that açai diet prevents BBB dysfunction in experimental CM.

### *Euterpe oleracea*-enriched diet decreases histological alterations in PbA-infected mice

**Fig. 4:**
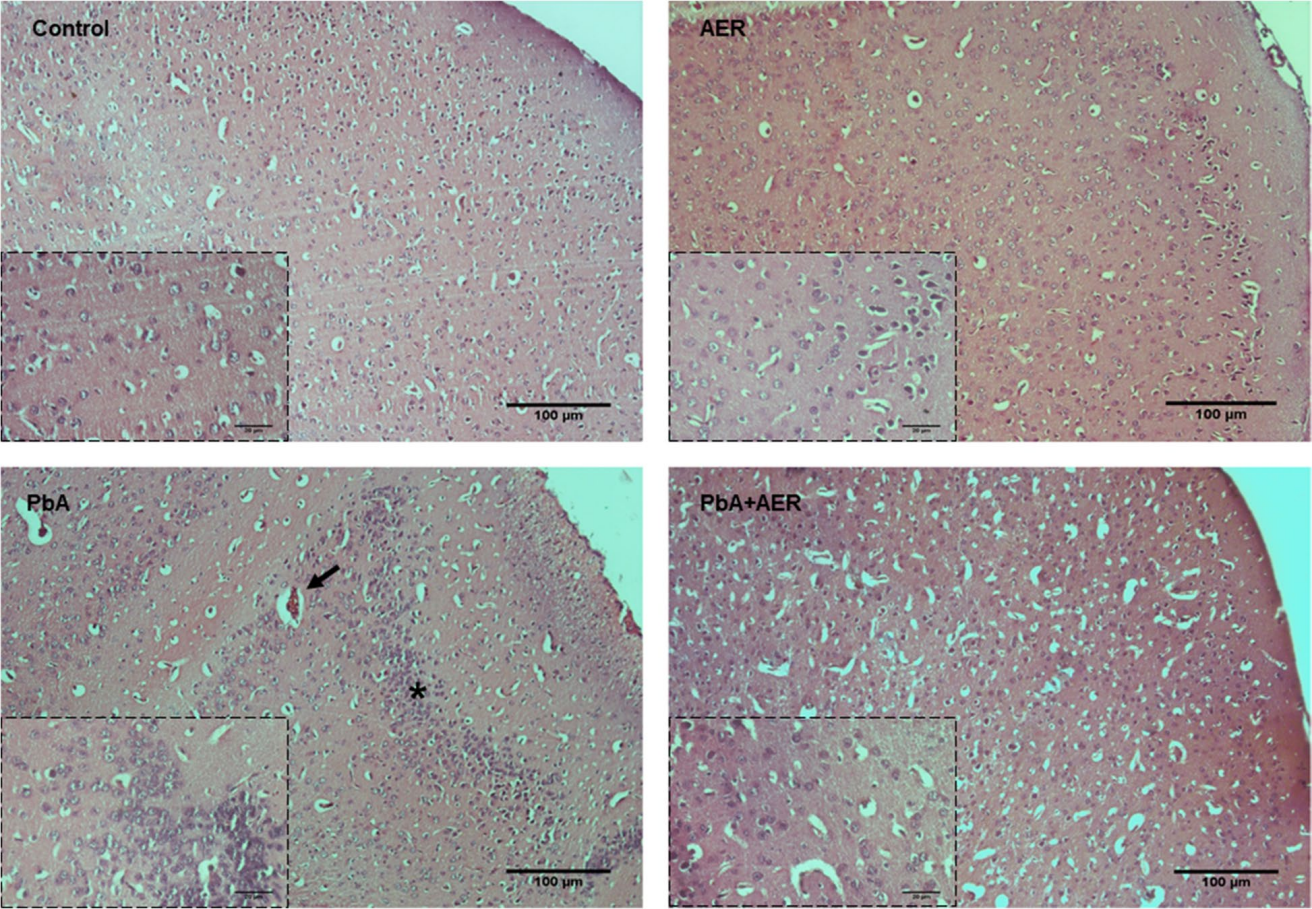
*Euterpe oleracea*-enriched diet decreases histological alteration into the brain tissue of PbA-infected mice. Representative light microphotographs from the cerebral cortex of mice at day 7 post infection and stained with HE (10x and 40x objetive lens). Brain cortex from uninfected mice (control group) with normal histological appearance and PbA-infected mice showing cellular infiltration (asterisk) and occluded capillaries (arrows)

At 7 days post infection, histological analysis of brain cortical slices exhibits a disarrangement in the cellular parenchyma indicating focal alterations such as inflammatory cell infiltrates, vascular dilatation and occluded capillaries (Fig. 4). In contrast, all these pathological changes were ameliorated in PbA-infected mice treated with açai-enriched diet (Fig. 4). In the cortical regions with severe inflammation, PbA-infected mice showed more diffuse and frequent cellular infiltration and occlusion compared to the brain of PbA-infected mice treated with açai-enriched diet.

### *Euterpe oleracea*-enriched diet attenuated neurobehavioral and motor impairments induced by PbA infection

**Fig. 5:**
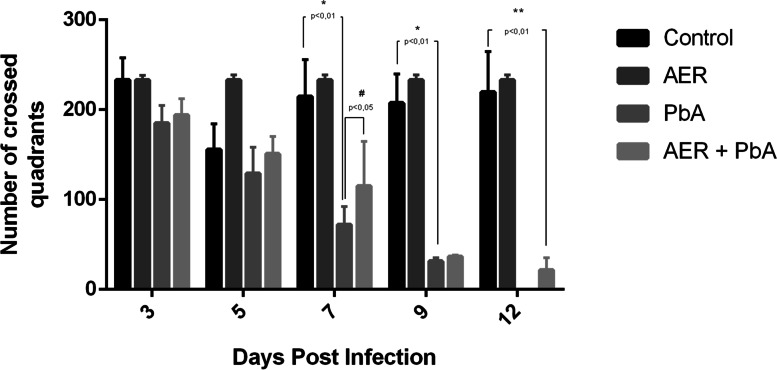
Protective effect of açai enriched diet against motor impairment induced by *Plasmodium berghei* ANKA infection. The total distance traveled was measured over 5 min in the open field test, based on the number of squares crossed on the 3rd, 5th, 7th, 9th and 12th day post-infection. *p < 0.01 (PbA vs. control), ^#^*p* < 0.05 (PbA vs. AER + PbA). Scores presented as the mean ± SEM

Open field test was performed to assess the effect of açai-enriched diet in locomotor activity of mice infected with PbA. As shown in Fig. 5, PbA-infected animals fed with standard diet presented significant decrease in mobility at 7- and 9-days post-infection. Otherwise, an increase in the total distance traveled in open field task was observed in PbA-infected mice treated with açai-enriched diet exhibiting an improvement in the overall locomotor activity at 7 days post infection, although this protective effect was not observed at 9th day post infection.

**Fig. 6:**
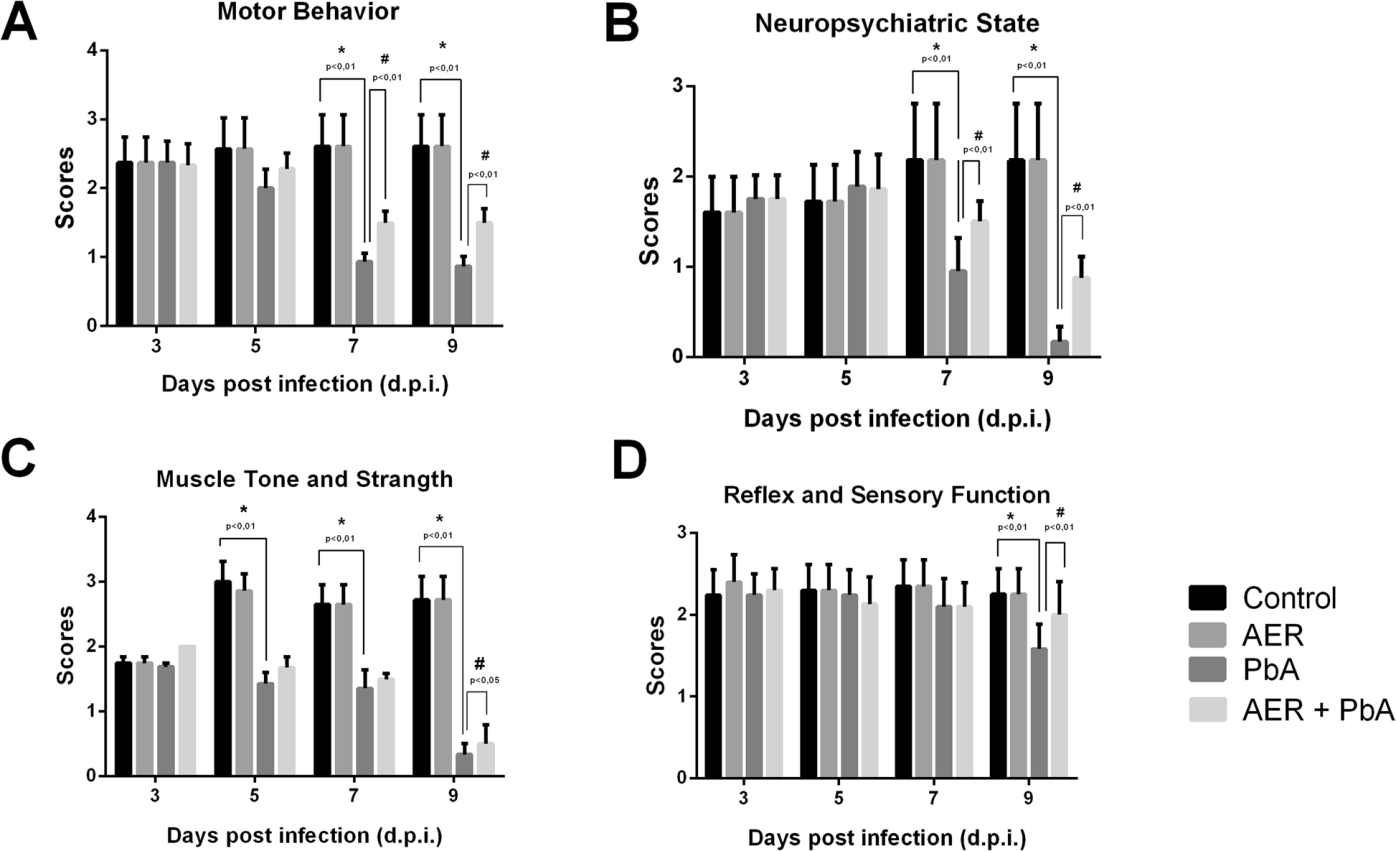
Clinical scores determined by SHIRPA protocol to assess neurobehavioral impairment elicited in swiss albino mice infected with *Plasmodium berghei* ANKA strain (PbA) treated with açai enriched ration. Four functional categories were evaluated: **A** motor behavior; **B** neuropsychiatric state; **C** muscle tone and strength and **D** reflex and sensorial function. Scores for all the categories were recorded as the general score for each group, with the data being presented as the mean ± SEM. The analyses were based on a one-way ANOVA, with Tukey’s post-test

Neurobehaviour impairments induced by CM were also assessed by distinct functional domains such as muscle tone and strength, motor behavior, neuropsychiatric capacity and reflex/sensorial state as described by SHIRPA protocol. Animals infected with PbA presented motor (Fig. 6B) and neuropsychiatric (Fig. 6C) impairments at 7th days post-infection, with açai-enriched diet providing protection against this deleterious cognitive impairment caused by CM.

At 9 days post-infection, data indicate PbA infection evokes a significant loss of all four domains analyzed, that was prevented by the treatment with açai-enriched diet which showed better scores when compared with PbA-infected animals fed with standard diet (Fig. 6). Taken together, data demonstrated that açai-enriched diet ameliorates clinical scores improving neurological signs of PbA-infected mice.

## Discussion

The present study demonstrated, for the first time, that an açai-enriched diet suppresses the development of CM in mice attenuating the breakdown of blood-brain barrier and the neurocognitive deficits associated with the disease. Usually, natural products are important and valuable sources of compounds with medicinal properties and diets enriched by these products are known to have a countless benefit to human health [28, 29]. Biochemical studies reveal the presence of phytochemicals compounds in these products that could modulate distinct signaling pathways implicated in several pathological conditions such as cancer, vascular disturbance, some neurodegenerative conditions as Parkinson and Alzheimer [30, 31] and infectious diseases such as leishmaniasis and malaria [32, 33].

Malaria is a very common infectious disease in children and adults living in tropical regions and many studies have already shown its deleterious effects in the CNS [2]. As motor and cognitive impairments are common neurobehavioral sequels in patients with CM [34], new adjuvant therapeutic interventions are extremely relevant to reduce neurological damages. Here, we reported a potential nutritional approach to protect the organism from the neuronal alterations associated to this condition. There is increasing evidence of potential neuroprotective properties of açai fruit, diminishing the neurodegenerative events associated with the progression of pathological conditions which affect the CNS [15, 35].

The pathophysiology of CM involves a range of cellular and histological processes, including cell sequestration, brain inflammation and the breakdown of the blood-brain barrier that can discharge major impairments in the behavior of infected animals [36]. The murine model of CM is a valuable tool to the development of effective treatments, given that mice reproduce most of the symptoms observed in humans [37]. In the present study, PbA-infected mice presented a number of symptoms characteristic of human cerebral malaria, including ataxia, poor reflexes, seizures and coma [36]. In animals fed with a normal diet (standard ration), neurological clinical signals of CM began to appear on 6-7th days after infection, succumbing to death between days 7 and 11 post infection, showing notable evidence of blood-brain barrier disruption, which confirms the development of CM in infected mice. Instead, animals treated with açai-enriched diet showed a significant improvement in the survival curve, with more than 20% of the group remaining alive until the 19th day post infection with no evidence of neurological impairments. Our work is in agreement with previous studies which demonstrated that the increase in survival rate is usually associated with improvements in neurological parameters [37, 38].

We also demonstrated that the neuroprotective effects of açai-enriched diet were not associated with changes in blood parasitemia of PbA-infected mice once the rate of parasitemia did not change in the course of the disease. Different from which we observed, Ferreira et al. [39] demonstrated that oral treatment by gavage with polyphenol-rich açai fractions reduced the parasitemia of *P. chabaudi-*infected mice, in an infection model in which there is no brain impairment. While recent studies have described antimicrobiotic activity of *Euterpe olarecea* fruit [40, 41], the results of the present study indicate that açai does not have antimalarial properties, and its protective effects are probably related to the attenuation of host response to the infection. In addition, our findings are in agreement with previous studies which demonstrated the activity of natural plant extract such as Zizyphus spina-christi and *Terminalia albida* against murine cerebral malaria [42, 43].

Besides delayed the outcome of neurological signals in PbA-infected mice, açai-enriched diet also prevented the breakdown of blood-brain barrier on the 7th day post infection. Furthermore, açai-enriched diet also attenuates brain cortex histological damages induced by PbA infection. Although our data showed that diet with açai enriched ration reduces the pathological changes in the brain with characteristic areas of leukocyte infiltration, additional studies need to be performed to elucidate this anti-inflammatory effect.

Disruption of the blood-brain barrier is known to be a consequence of two main processes induced by *Plasmodium* infection. First, brain vessels are obstructed by parasitized erythrocytes, evoking local hypoxia and intense oxidative stress [44]. Second, the infection can also stimulate an inflammatory response in the brain, causing overproduction of pro-inflammatory cytokines and reactive oxygen species (ROS) [45, 46]. Those events are followed by the degradation of endothelial cells, which damages the blood-brain barrier [47, 48]. A number of studies have demonstrated that the chemical composition of açai fruit confers it with valuable antioxidant and anti-inflammatory properties, and it seems likely that these compounds produce a protective effect in the brain of the PbA-infected mice fed with açai-enriched ration [49–52]. While additional studies need to be done to ratify this hypothesis, several works have shown that oral intake of açai decreases the level of IL-1β, IL-18 and TNF-α, prevents the production of thiobarbituric acid reactive substance (TBARS) and modulates antioxidant enzymes such as superoxide dismutase (SOD) and catalase [53].

Some studies attribute the anti-inflammatory and antioxidant effect of açai to the high levels of anthocyanins and other polyphenols (flavonoids) in its composition [47, 54, 55]. It is well described that the beneficial effects of açai can be attributed to several bioactive molecules, but its antioxidant activity is often associated with the phenolic compounds, among which orientin, homoorientin, catechin, epicatecin, ferrulic acid, vanillic acid, gallic acid, p- hydroxybenzoic acid and syringic acid. The phenolic compounds contribute to maintain the balance between the production and elimination of oxygen reactive species, thus reducing cell damage caused by free radicals [18, 56]. Although additional studies are required to elucidate the mechanisms by which açai diet exerts its neuroprotective effect in PbA infected mice, we suggested that phenolic content contributes to this effect.

The breakdown of blood-brain barrier is often associated with behavioral dysfunctions in the CNS [57, 58]. A number of studies have already demonstrated that *Plasmodium falciparum* infection triggers long-term neurological deficits such as cognitive impairment, motor skills, visual acuity and seizures [59, 60]. Events associated with inflammation and oxidative stress could also be related to these deficits [61, 62]. In our work, we demonstrated, for the first time, that açai-enriched diet improved sensorimotor outcomes related to CM condition.

The results of the present study indicate that açai-enriched diet has a relative protective action on peripheral behavioral parameters such as motor performance, muscle tone and strength. However, açai-enriched diet has a more effective preventive action on the impairments in neuropsychiatric and sensorial function induced by PbA infection. Overall, the results of the present study indicate that açai intake was more effective in the protection of CNS impairments than in the motor system.

Thus, this preclinical study demonstrated that açai intake may minimize the deleterious effects of CM on the brain of PbA-infected animals, and an açai-enriched diet may be a potentially valuable adjuvant for the treatment of human cerebral malaria. Moreover, to further elucidate the mechanism by which açai-enriched diet exert its neuroprotective role, molecular and biochemical approaches should be performed to address the anti-inflammatory and antioxidant effects of the diet.

## Conclusion

The present study demonstrates a significant and potent neuroprotective effect of açai enriched diet in the outcome of cerebral malaria condition, protecting the host from the blood-brain barrier disruption and neurobehavioral impairments associated with the disease. Although the mechanism of this neuroprotective effect is not fully understood, it is probably due to the anti-inflammatory and antioxidant effects of açai compounds.

## Data Availability

Authors declare that data supporting the findings of this study are available in a public database (Mendeley Data; 10.17632/hc785xkvfn.1).
